# Alkaline fluid circulation in ultramafic rocks and formation of nucleotide constituents: a hypothesis

**DOI:** 10.1186/1467-4866-7-7

**Published:** 2006-07-25

**Authors:** Nils G Holm, Marion Dumont, Magnus Ivarsson, Cécile Konn

**Affiliations:** 1 Dept. of Geology and Geochemistry, Stockholm University, Stockholm, Sweden

## Abstract

Seawater is constantly circulating through oceanic basement as a low-temperature hydrothermal fluid (<150°C). In cases when ultramafic rocks are exposed to the fluids, for instance during the initial phase of subduction, ferromagnesian minerals are altered in contact with the water, leading to high pH and formation of secondary magnesium hydroxide, among other – brucite, that may scavenge borate and phosphate from seawater. The high pH may promote abiotic formation of pentoses, particularly ribose. Pentoses are stabilized by borate, since cyclic pentoses form a less reactive complex with borate. Analyses have shown that borate occupies the 2' and 3' positions of ribose, thus leaving the 5' position available for reactions like phosphorylation. The purine coding elements (adenine, in particular) of RNA may be formed in the same general hydrothermal environments of the seafloor.

## Background

Oceanic basement consists of basalts and ultramafic rocks that have relatively low silica contents (45–52% and <45%, respectively) but a high content of ferromagnesian minerals like olivine and pyroxene. Alteration of these minerals in contact with water during free hydrothermal circulation leads to 'serpentinization', a process in which olivine reacts with water. This may lead to the formation of serpentine, magnetite, brucite, and molecular hydrogen [1]. The process may also be associated with high alkalinity [2]. Alkaline fluids are characteristic of deep aquifers of ultramafic rocks such as the Oman ophiolite (pH 10–12 [3]), the Coast Range ophiolite (pH 11–12 [4]), as well as hydrothermal systems of ridge flanks (Lost City; pH 9–9.8 [5,6]) and non-accretionary suprasubduction zones (Mariana forearc; pH 12.6 [7]). Serpentinite-hosted hydrothermal vent fields appear to be common along slow and ultraslow spreading ridges [8,9].

Brucite is a single-layer magnesium hydroxide mineral that may be transformed into double-layer hydroxides if a fraction of the divalent Mg^2+ ^is replaced by common trivalent cations such as Al^3+^, Fe^3+ ^and Cr^3+ ^[10,11]. Magnetite is an efficient catalyst in Fischer-Tropsch type (FTT) reactions and the abiotic synthesis of organic compounds [12,13]. Different classes of mostly linear organic compounds are formed in FTT reactions from H_2 _and CO or CO_2 _in the presence of mixtures of native transition metals or their oxides. The type of organic compound formed depends on the catalyst or mixture of catalysts present. Geochemists often refer to the FTT reaction pathways for the reduction of CO_2 _to CH_4 _in aqueous environments even though the industrial term normally covers only the reduction of CO to a variety of organic compounds under anhydrous conditions [1,14-16]. Experimental data by Berndt and co-workers suggested that magnetite's catalyzing effect on FTT synthesis is maintained during reaction under high water pressures [17]. Subsequent studies by McCollom and Seewald have shown that their interpretation was probably not correct, and that ethane and propane do not form from FTT processes under the conditions used by Bernd et al. [18]. However, Foustoukos and Seyfried have found that abiotic formation of hydrocarbons in hydrothermal fluids is promoted by a mix of iron- and chromium-bearing minerals [19]. These results may suggest that the chromium component in ultramafic rocks is an important factor for FTT synthesis under hydrous conditions.

In addition, Madon and Taylor have shown that magnetite is much less susceptible to poisoning by compounds such as H_2_S than metallic iron, and therefore, is efficient under a wide range of conditions in natural environments [20].

## Ribose and the formose reaction

A couple of decades ago many scientists believed that the formation of ribose, a constituent of RNA, occurred through the formose reaction [21,22]. In this reaction, pentoses like ribose can be formed under alkaline conditions from simple organic precursors (formaldehyde and glycolaldehyde) [22,23]. The condensation of formaldehyde to sugars is catalyzed by divalent cations and layered minerals, such as clays. The reaction proceeds by the stepwise condensation of formaldehyde to a dimer (glycolaldehyde), trimer, etc. Under experimental conditions it has been possible to convert as much as 50% of the original formaldehyde to glycolaldehyde [24]. However, this reaction has for a while been an outdated concept in prebiotic chemistry. A major reason for this is that the reaction, as we have known it, is nonselective and leads to a large variety of aldoses, ketoses, and sugar alcohols with only small fractions of potentially bioactive compounds such as ribose [25-27]. A general opinion has been that if ribose were used in the first RNA, an unknown selection process must have operated to segregate ribose from the other sugars that were formed. A second reason why the formose reaction has been outdated is that the reaction proceeds at a constructive rate only under naturally 'improbable' conditions, like under highly alkaline conditions [11,21,22,28]. Natural environments with the pH conditions required for the abiotic formation carbohydrates have previously been considered to be relatively rare on Earth. However, the recent discovery of alkaline hydrothermal systems in ultramafic rocks, like the Lost City Hydrothermal Field on the Mid-Atlantic Ridge [5,6], indicates that alkaline environments may be much more common on Earth than we thought just a few years ago. Therefore, the formose reaction must still be considered to be of great potential if we could identify some selective mechanism that would interact with it. Below, we propose that such a mechanism is known today.

## Pentoses are stabilized by borate

**Figure 1:**
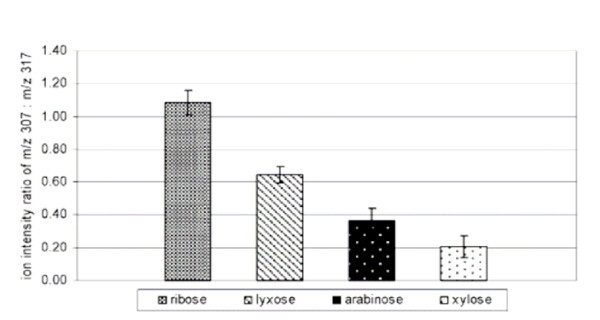
The preferential order of binding of pentoses to boron is ribose>lyxose>arabinose>xylose. Reprinted with permission from Li et al. [33]. Copyright 2005 American Chemical Society.

It has recently been shown that borate minerals stabilize ribose [29,30]. Both boric acid and borate readily form complexes with a wide variety of sugars and other compounds containing cis-hydroxyl groups [31,32]. Once formed, the cyclic form of the pentose like ribose forms a stable, less reactive complex with borate. The binding preferences of borate to pentoses has been determine to be ribose>lyxose>arabinose>xylose (Fig. 1) [33]. NMR analysis shows that borate occupies the 2' and 3' positions of ribose, thus leaving the 5' position available for potential reactions like phosphorylation [30,34]. In biological systems, the purine nucleotides are synthesized by constructing the purine base on a pre-existing ribose-5'-phosphate [21]. However, results by Etaix and Orgel show that adenosine-5'-triphosphate can be synthesized directly from adenosine and trimetaphosphate if the 2'- and 3'-OH groups are blocked by borate [35]. On the other hand, even though Yamagata and coworkers have found trimetaphosphate in fumaroles of Mount Usu, Japan [35], it is perhaps not the most likely phosphorylating agent under natural conditions. Yamagata and coworkers also identified about equal concentrations of pyrophosphate in the fumaroles (0.45 μM) [35]. Pyrophosphate is a phosphorus compound that appears to form under more varied conditions and is, therefore, probably a more likely candidate for abiotic phosphorylation (see below).

## Purines and amino acids may be formed in the same prebiotic environments

Unlike ribose, the purine coding elements of RNA can be synthesized in the same abiotic reactions that yield amino acids [22,23,26,37]. Amino acids may be synthesized in putative prebiotic chemistries like Strecker type reactions (synthesis of amino acids from cyanide and aldehyde in the presence of ammonia) in hydrothermal environments at fairly low temperatures (150°C) [38]. Amino acids can also be released by hydrolysis of HCN oligomers that form by the self-condensation of hydrogen cyanide in aqueous solution [23]. Such reactions do not require alkaline conditions. Purines are formed from HCN via two routes. One route is via the HCN oligomers that also forms amino acids; the second one is via the HCN tetramer diaminomaleonitrile (DAMN) [22,23]. HCN may be formed in a variety of ways but normally occur in trace amounts. In order to participate in abiotic organic reactions they must first be concentrated. One possibility is concentration to a reservoir of iron cyanide at relatively low pH from which free HCN can be released upon local elevation of the pH [11]. This would avoid the 'Miller paradox', which refers to the side reaction of stable cyanohydrin formation from free HCN and ubiquitous formaldehyde. Russell and coworkers have presented a model involving alkaline hydrothermal mounds as flow reactors in which strongly polar compounds such as the cyanide ion is retained by fresh FeS/Fe_3_S_4 _membranes [2]. According to their model, the fluctuations in pH at the interface between hydrothermal fluid and seawater would determine adsorption and desorption of the cyanide. In natural environments, the occurrence of ferrocyanides in hydrothermal systems has so far been reported from the Kurile Islands and the Kamchatka Peninsula [39,40].

The self-condensation of HCN to produce purines is a simple and efficient reaction [22]. Joyce has, therefore, suggested that the first genetic material was based on purine bases alone [21]. The hypothesis has been supported by experimental results of Sowerby and coworkers [41]. Their experiments showed that both adenine and hypoxanthine that was adsorbed on graphite surfaces modulated the interaction of amino acids with the crystal surface. Adenine and hypoxanthine are the coding elements of a putative purine-only genetic alphabet and the observed effects on amino acids were different for each of the bases. However, Cohn and coworkers have shown that adenine is far displaced toward adsorption onto pyrite, quartz and pyrrhotite, which are all common minerals of hydrothermal environments [42]. It would, therefore, normally be useless to search for the purine bases in the fluid phase of hydrothermal systems [43].

## Aldehydes in hydrothermal systems

Due to the postulated difference in requirements for the formation of the ribose and the nitrogen base, the spontaneous formation of RNA under prebiotic conditions has been doubted [26]. The differences in required environment may, however, be illusive. We mentioned before that formaldehyde is necessary for the formation of carbohydrates in the formose reaction. Schulte and Shock have shown that aldehydes may be intermediates in the formation of carboxylic acids from hydrocarbons in sedimentary basin brines as well as in hydrothermal systems [44]. Furthermore, they concluded that the presence of aldehydes should normally be difficult to detect in natural systems if metastable equilibrium is reached between aldehydes and carboxylic acids at expected redox conditions. On the other hand, this suggests that aldehydes are always present as reaction intermediates if organic acids and hydrocarbons exist in natural hydrothermal systems. In fact, low concentrations of formaldehyde have been identified in hot spring environments in Iceland, Mexico and Southern California [45].

## The Mariana forearc

The Mariana forearc in the western Pacific Ocean, where the Pacific plate subducts beneath the Philippines plate, seems to be of particular interest in the context of abiotic organic synthesis. The Mariana forearc is a non-accretionary forearc with numerous seamounts. The sedimentation rate of pelagic material is very slow and the carbon content of the serpentinite mud on the seafloor is extremely low (0.01–0.1 wt.%) [7]. 'Contamination' by biogenenic input into the system is thus at minimum. Interstitial fluids of pH 12.5 associated with serpentinized mud at the South Chamorro seamount are enriched in dissolved carbonate, light hydrocarbons, borate and ammonia [7]. Pore fluids from Conical Seamount contain light hydrocarbons as well as organic acids, while fluid inclusions of the associated carbonate chimneys show the presence of light as well as longer chain hydrocarbons, aromatics and acetate [46]. These fluids derive from the subducting Pacific plate at an early stage of dehydration. After the fluids have been expelled from the subducting plate at moderate temperatures they are cooled down to a few °C on passage through the overriding Philippines plate/Mariana forearc. The unusually high δ^13^C of the methane present in the Mariana forearc fluids and the relatively low C1/C2 ratio suggest an abiotic origin of the carbon compounds [7].

Seawater is the source of virtually all of the borate in altered oceanic crust. Boron is rapidly taken up from seawater during low-temperature alteration of the oceanic crust [47,48]. Boron is conspicuously enriched in serpentinites and basalt altered by seawater at relatively low temperatures [49,50]. At 150°C and below boron is removed from the seawater and is incorporated into brucite, which is the dominant alteration phase. It has been shown that boric acid inhibits the dissolution of brucite at neutral and weakly alkaline pH [51].

## Brucite scavenges phosphate

Co-precipitation with brucite at high pH is used analytically for quantitative removal and the precise determination of nanomolar concentrations of phosphate in natural fluids [52]. This is one of the few natural mechanisms to concentrate phosphate relative to ambient conditions. Also, Al-substituted Mg-hydroxide double layer minerals have shown the intercalation of, for instance, phosphate, sugar-, aldol-, and alkyl phosphates and nucleotides [53].

Solid magnesium hydroxides with adsorbed phosphate have, furthermore, been shown to catalyze the synthesis of pyrophosphate from orthophosphate [54]. Hermes-Lima and Vieyra in their article claim that they synthesize magnesium phosphate, although this is not verified by, for instance, x-ray analysis. However, the method they specify involves high pH [55], which suggests that they produce brucite [56], in analogy to the procedure of Karl and Tien [52], with phosphate being co-precipitated. The formation of pyrophosphate was shown to be most efficient above pH 9. Pyrophosphate that is formed in such a way would stay concentrated on the magnesium hydroxide after 'activation' of the adsorbed orthophosphate [54], potentially together with an abiotic purine nucleoside. Once pyrophosphate is available, phosphorylation of the nucleoside is possible.

Krishnamurthy and coworkers have found that glycolaldehyde is converted to glycolaldehyde phosphate (GAP) in the presence of amidotriphosphate [57]. The conversion is complete only in the presence of 0.25 M MgCl_2_. They concluded that 'the role of magnesium ion, while essential for the reaction to proceed, is not easily specified in detail'. The presence of magnesium is obviously important for the progress of phosphorylation reactions in natural environments, but we do not yet know why.

## Ferroan brucite – an intermediate on the way to brucite and magnetite

Recent work by Bach and coworkers suggests that serpentinization goes through a sequence of reactions that start with low fluid flux serpentinization of olivine to serpentine and ferroan brucite [58]. Later-stage serpentinization invokes formation of magnetite and brucite by the breakdown of the ferroan brucite [58]. This means that phosphate (orthophosphate and pyrophosphate) and borate that is scavenged by the brucite will be in close contact with the authigenic magnetite while it is being formed as a microcrystalline compound with a large surface area and, presumably, is most potent for the abiotic formation of organic compounds.

## The entire ocean floor is affected by fluids

**Figure 2:**
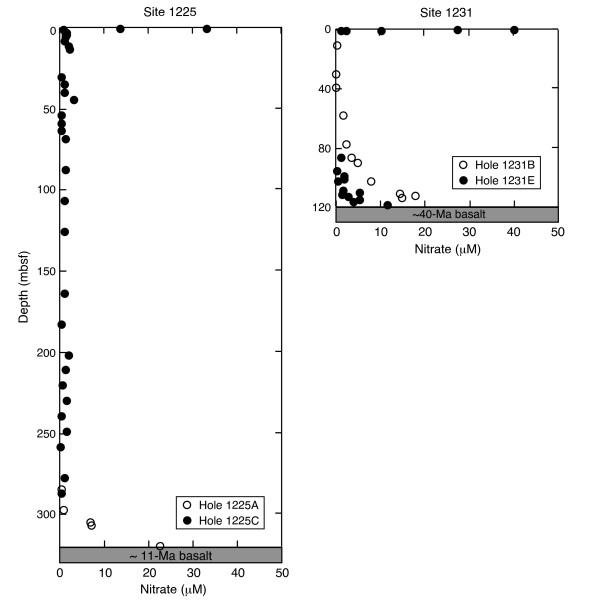
Dissolved nitrate concentrations in sediment pore fluids at open-ocean ODP Sites 1225 and 1231. The nitrate values show that fresh seawater is channelled upwards into deep-sea sediments via the rocks underneath (from D'Hondt et al., 2003 [59]).

The Ocean Drilling Program (ODP) Leg 201 was devoted to the controls on microbial communities in deeply buried sediments and was carried out in 2002. Results from ODP Leg 201 reveal that fresh seawater is channelled upwards into deep-sea sediments from the rocks underneath [59,60]. This happens still 40 Ma or more after formation of the basement and is illustrated by the concentration profiles of dissolved nitrate in sediment porewater from ODP Sites 1225 and 1231 (Fig. 2). Similar profiles have been obtained for dissolved sulfate. Such fluid flow must, therefore, be considered a global process that occurs over a wide range of temperatures. The fact that seawater circulates through ocean basement millions of years after its formation shows that hydrothermal processes at moderate temperatures can be quite extended in time. This, together with the recent discoveries of mechanism of serpentinization and abiotic organic synthesis, adds a dimension of universatility to possible scenarios for the prebiotic formation of the first genetic material.

## Conclusion

It is, indeed, possible that the formose reaction is responsible for the prebiotic formation of ribose in natural environments and that this occurs in close vicinity to purine synthesis and phosphorylation processes. Shapiro a couple of decades ago concluded: 'The evidence that is currently available does not support the availability of ribose on the prebiotic Earth... This situation could change if some alternative pathway for ribose synthesis were discovered; one that produced it in better yield and was not as vulnerable to interferences from nitrogen-containing substances' [26]. The discovery of the stabilization of pentoses – ribose, in particular – by borate has now changed our view of the formose reaction from a seemingly random and nonselective reaction into a very precise pre-RNA process.
